# Inhibition of the activation of γδT17 cells through PPARγ–PTEN/Akt/GSK3β/NFAT pathway contributes to the anti-colitis effect of madecassic acid

**DOI:** 10.1038/s41419-020-02969-x

**Published:** 2020-09-14

**Authors:** Xinming Yun, Yulai Fang, Changjun Lv, Simiao Qiao, Yu Tao, Yue Dai, Yufeng Xia

**Affiliations:** 1 Department of Pharmacognosy, School of Traditional Chinese Pharmacy, China Pharmaceutical University, 24 Tong Jia Xiang, 210009 Nanjing, PR China; 2 Department of Pharmacology of Chinese Materia Medica, School of Traditional Chinese Pharmacy, China Pharmaceutical University, 24 Tong Jia Xiang, 210009 Nanjing, PR China

**Keywords:** Pharmacodynamics, Acute inflammation

## Abstract

Type-17 immune response, mediated mainly by IL-17, plays a critical role in ulcerative colitis. Previously, we showed that madecassic acid (MA), the main active ingredient of *Centella asiatica* herbs for anti-colitis effect, ameliorated dextran sulfate sodium (DSS)-induced mouse colitis through reducing the level of IL-17. Here, we explore the effect of MA on the activation of γδT17 cells, an alternative source of IL-17 in colitis. In DSS-induced colitis mice, oral administration of MA decreased the number of γδT17 cells and attenuated the inflammation in the colon, and the anti-colitis effect of MA was significantly counteracted by redundant γδT17 cells, suggesting that the decrease in γδT17 cells is important for the anti-colitis effect of MA. In vitro, MA could inhibit the activation but not the proliferation of γδT17 cells at concentrations without evident cytotoxicity. Antibody microarray profiling showed that the inhibition of MA on the activation of γδT17 cells involved PPARγ–PTEN/Akt/GSK3β/NFAT signals. In γδT17 cells, MA could reduce the nuclear localization of NFATc1 through inhibiting Akt phosphorylation to promote GSK3β activation. Moreover, it was confirmed that MA inhibited the Akt/GSK3β/NFATc1 pathway and the activation of γδT17 cells through activating PPARγ to increase PTEN expression and phosphorylation. The correlation between activation of PPARγ, decrease in γδT17 cell number, and amelioration of colitis by MA was validated in mice with DSS-induced colitis. In summary, these findings reveal that MA inhibits the activation of γδT17 cells through PPARγ–PTEN/Akt/GSK3β/NFAT pathway, which contributes to the amelioration of colitis.

## Introduction

Ulcerative colitis (UC) is a chronic relapsing–remitting inflammatory disease with a high risk of developing colorectal cancer in long-term patients^1^. The greatest increase in the incidence of UC is observed in newly industrialized countries, and the highest incidence and prevalence is recorded in Western countries, particularly in Europe and America^2^. Although the etiology of UC remains unclear, studies in recent years emphasize the importance of the dysregulation of immune responses, especially type-17 immune response, in the initiation, augmentation, and perpetuation of UC^3,4^. The type 17 immune response is mediated mainly by the activation of IL-17-producing cells, including αβT cells, γδT cells, and innate lymphoid cells (ILCs)^5–7^. The signature cytokines of type-17 immune response include IL-17, IL-21, IL-22, IL-23, IL-6, TNF-α, GM-CSF, and so on^8,9^, among which IL-17 is closely associated with the development and progression of UC^10^. Therefore, reducing the level of IL-17, produced by type-17 immune cells, is a practical therapeutic strategy for treating UC.

Madecassic acid (MA), a triterpenoid constituent in *Centella asiatica* herbs, has anti-inflammatory^11^, antidiabetic^12^, anti-oxidant^13^, neuroprotective^14^, and anticancer effects^15^. In our previous studies^16^, MA, orally administered, was shown to obviously attenuate DSS-induced colitis in mice. MA could reduce the expression of RORγt and IL-17 in the colon tissues of mice, suggesting that the anti-colitis mechanism is probably associated with the downregulation of the IL-17 level in colon tissue. However, under in vitro Th17-polarizing condition, MA only weakly decreased the expression of RORγt and IL-17, implying that other IL-17-producing cells are involved in the anti-colitis effect of MA. Accumulative evidence indicates that γδT cells are the alternative contributors of IL-17 in addition to CD4^+^αβT cells in type-17 immune response of colitis^4,17–19^. The IL-17-producing γδT (γδT17) cells might be the target cells of MA for the anti-colitis effect.

This study aims to identify the effect of MA on the activation of γδT17 cells and the relation to the anti-colitis effect. The underlying mechanism of MA is also explored.

## Results

### γδT17 cells are the main target cells of MA for reducing the expression of IL-17 in the colon of colitis mice

**Fig. 1:**
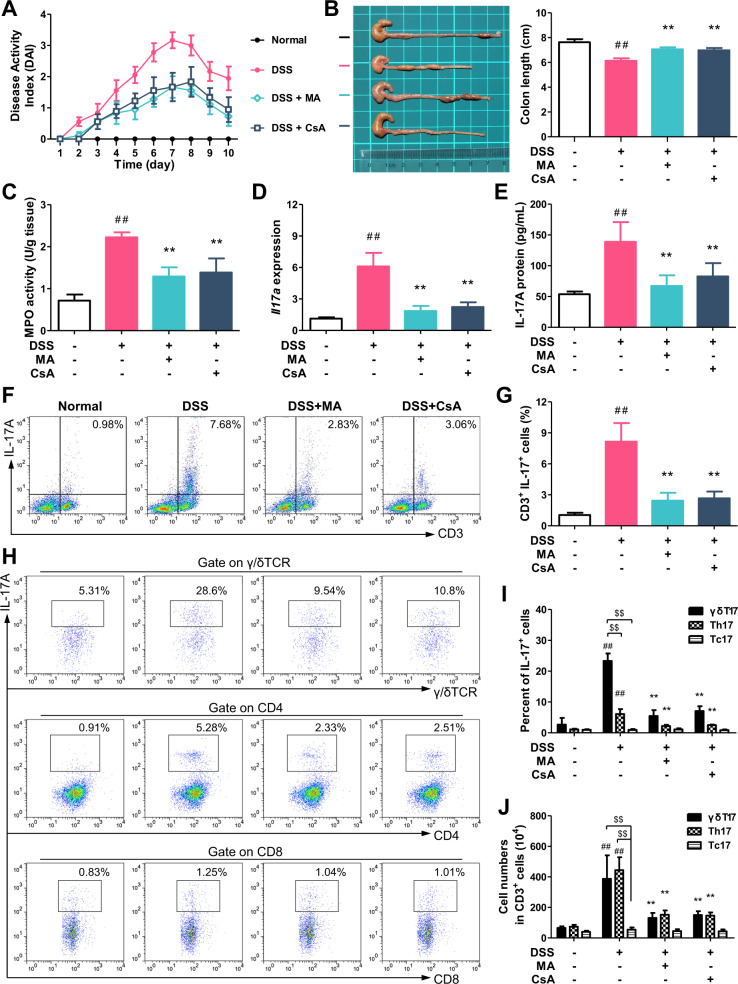
Madecassic acid decreases the number of γδT17 cells in the colons of mice with dextran sulfate sodium (DSS)-induced colitis. Colitis was induced in female C57BL/6 mice by drinking with 2.5% DSS for 7 days, and followed by normal drinking water for 3 days. Madecassic acid (MA, 25 mg/kg) and cyclosporin A (CsA, 25 mg/kg) were orally gavaged for 10 days consecutively. **a** Disease activity index (DAI). **b** Colon length. **c** Myeloperoxidase (MPO) activity. **d** The expression of *Il17a* mRNA as assessed by real-time PCR. **e** The levels of IL-17A as assessed by enzyme-linked immunosorbent assay. **f**, **g** The representative flow cytometry and percentage of CD3^+^IL-17^+^ cells in the mononuclear cells of lamina propria from mouse colon tissues. **h** The representative flow cytometry of IL-17 expression in CD8^+^ T cells (Tc17), CD4^+^ T cells (Th17), and γδTCR^+^ T cells (γδT17) in CD3^+^ cells. **i** The percentages of IL-17A^+^ subpopulations present in the CD8^+^, CD4^+^, and γδTCR^+^ T cells. **j** The absolute number of Tc17, Th17, and γδT17 cells in CD3 + cells (10^4^). The data are expressed as means ± SEM of six mice per group. ^##^*P* < 0.01 versus the normal group; ^*^P < 0.05, ^**^P < 0.01 versus the DSS group, ^$$^*P* < 0.01 versus the Tc17 cell group.

Colitis was induced in female C57BL/6 mice by drinking with 2.5% DSS for 7 days, and followed by normal drinking water for 3 days. MA (25 mg/kg/day) and cyclosporin A (CsA, 25 mg/kg/day), as positive control, were orally gavaged for 10 days. The mice treated with DSS exhibited weight loss, diarrhea, and blood, and an elevated cumulative disease activity index (DAI). On the 10th day, all mice were sacrificed. The DSS-treated mice showed shorter colons, higher activity of myeloperoxidase (MPO), and promoted expression of *Il17a* and IL-17A in the colon tissues. Consistent with our previous report^16^, orally administered MA and CsA showed significant ameliorative effect on colitis, and reduced the expression of IL-17 in colon tissues (Fig. 1a–e). The anti-colitis effects of MA and CsA showed no significant difference.

To identify the target cells of MA for reducing the expression of IL-17 in the colon of colitis mice, single-cell suspensions were prepared from the colon tissues and analyzed by flow cytometry. The data showed that CD3^+^ cells were the main IL-17-producing cells, and the percentage of CD3^+^IL-17^+^ cells was significantly decreased by MA treatment (Fig. 1f, g). In the colon tissues, CD3^+^IL-17^+^ cells mainly include Th17, Tc17, and γδT17 cells. The numbers of Th17 and γδT17 cells significantly increased in the lamina propria of the colons of DSS-treated mice, and the absolute numbers of γδT17 and Th17 cells were far more than Tc17 cells. MA treatment markedly decreased the absolute numbers of γδT17 and Th17 cells, but not Tc17 cells (Fig. 1h, j). Moreover, the percentage of γδT17 cells was more greatly decreased than Th17 cells with the treatment of MA (Fig. 1h, i). In combination with our previous findings that MA hardly affects the generation of Th17 cells^16^, we speculate that γδT17 cells are the main target cells of MA for reducing IL-17 expression in colon tissues of colitis mice.

### Decrease in γδT17 cell number plays a critical role in MA ameliorating DSS-induced mouse colitis

**Fig. 2:**
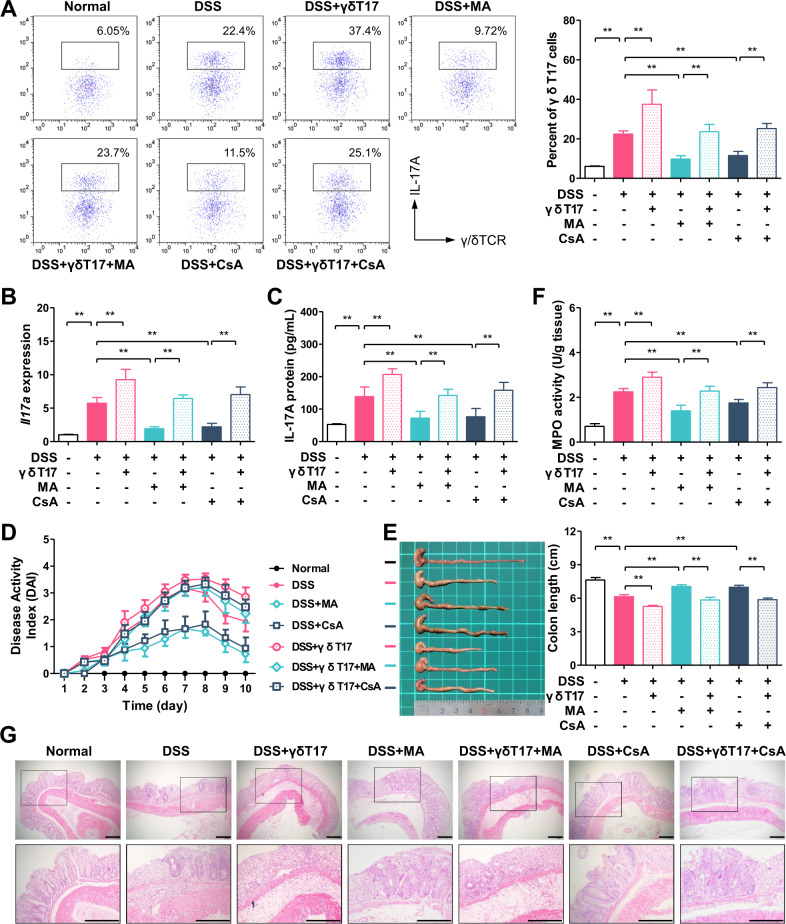
Anti-colitis effect of madecassic acid is counteracted by redundant γδT17 cells. The γδT17 cell rescue experiment was performed. The γδT cells were stimulated with IL-1β (10 ng/mL) and IL-23 (10 ng/mL) for 72 h; then, they were adoptively transferred into the recipient mice (10^5^ cells/mouse in 200 μL of PBS) on the 3rd and 7th days. The same volume of PBS was injected as the control. **a** The representative flow cytometry and percentages of IL-17A^+^ subpopulations present in the γδTCR^+^ T cells from mouse colon tissues. **b** The expression of *Il17a* mRNA as assessed by real-time PCR. **c** The levels of IL-17A as assessed by enzyme-linked immunosorbent assay. **d** Disease activity index (DAI). **e** Colon length. **f** Myeloperoxidase (MPO) activity. **g** Hematoxylin and eosin (H&E) staining (scale bar, 100 μm). The data are expressed as means ± SEM of six mice per group. ^*^P < 0.05, ^**^P < 0.01 versus the indicated group.

To recognize if the decrease in γδT17 cell number is critical for the anti-colitis effect of MA, an adoptive transfer experiment was performed. The γδT cells, purified from the spleens of normal mice, were activated by stimulation with IL-1β and IL-23, and were then adoptively transferred to mice with colitis. As expected, the transfer of activated γδT17 cells led to an increased number of γδT17 cells in the lamina propria of mouse colons (Fig. 2a), and an elevated expression of *Il17a* and IL-17A (Fig. 2b, c). Accordingly, colitis was aggravated by the transfer of γδT17 cells, evidenced by higher DAI, shorter colon length, higher MPO activity, and worse pathological lesion in the colon tissues (Fig. 2d–g). The anti-colitis effects of MA and CsA (a selective inhibitor for the activation of T cells) were markedly diminished in the mice transferred with activated γδT17 cells (Fig. 2d–g), suggesting that the reduction of the number or activation of γδT17 cells plays a critical role in MA ameliorating DSS-induced colitis in mice.

### Madecassic acid inhibits the activation of γδT17 cells in vitro

Unlike conventional αβT cells that differentiate into effector cells when encountered with stimulus signals in peripheral tissues, γδT effector subsets were functionally preprogrammed during ontogeny in the thymus before their distribution to the periphery^20–22^. Therefore, the detectable γδT17 cell number represents the activation extent of γδT17 cells in colon tissues.

**Fig. 3:**
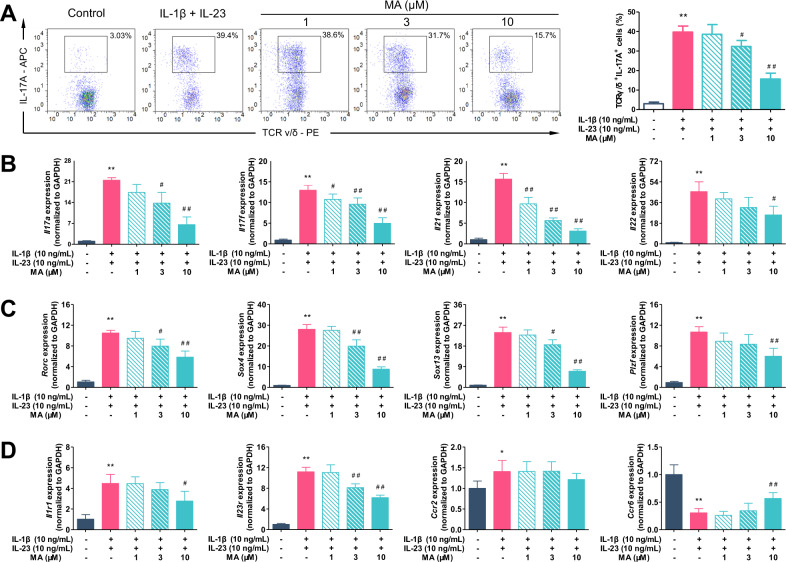
Madecassic acid inhibits the activation of γδT17 cells in vitro. The purified γδT cells were cultured with IL-1β (10 ng/mL) and IL-23 (10 ng/mL) for 72 h in the presence of madecassic acid (MA, 1, 3, or 10 μM). **a** The representative flow cytometry and percentages of IL-17A^+^ subpopulations present in the γδTCR^+^ T cells. **b** The expression levels of the cytokines in γδT17 cells, including *Il17a*, *Il17f*, *Il21*, and *Il22*, as assessed by real-time PCR. **c** The expression levels of the transcription factors of γδT17 cells, including *Rorc*, *Sox4*, *Sox13*, and *Plzf*, as assessed by real-time PCR. **d** The expression levels of the surface receptors of γδT17 cells, including *Il1r1*, *Il23r*, *Ccr2*, and *Ccr6*, as assessed by real-time PCR. The data are expressed as means ± SEM from three independent experiments. ^*^P < 0.05, ^**^P < 0.01 versus the untreated group; ^#^*P* < 0.05, ^##^*P* < 0.01 versus IL-1β and the IL-23-stimulated group.

To evaluate whether MA can regulate the activation of γδT17 cells, the γδT cells were purified from the spleen cells of mice by magnetic-activated cell sorting (MACS) (Supplementary Fig. S1). In vitro, the γδT cells were stimulated with IL-1β and IL-23, and then exposed to different concentrations (1, 3, and 10 μM) of MA for 72 h. The results showed that MA (3 and 10 μM) decreased the percentage of TCRγ/δ^+^IL-17^+^ T cells (Fig. 3a). Consistently, MA (3 and 10 μM) downregulated the expression of cytokines, including *Il17a*, *Il17f*, *Il21*, and *Il22*, which were produced from γδT17 cells under the stimulation of IL-1β and IL-23 (Fig. 3b). The expression of γδT17 cell-specific transcription factors, such as *Rorc*, *Sox4*, *Sox13*, and *Plzf*, was also reduced by MA treatment (Fig. 3c). In addition, MA lowered the expression of *Il1r1* and *Il23r*, and enriched the expression of *Ccr6* without significant impact on the expression of *Ccr2* (Fig. 3d), resulting in the lower sensitivity of γδT17 cells to cytokine stimulation. In order to eliminate the interference of cytotoxicity and proliferation, MTT and proliferation assays were performed. The results showed that MA had no obvious effect on the viability of γδT cells at concentrations lower than 30 μM for 72 h, and did not influence the expansion of γδT cells at concentrations lower than 30 μM for 2 weeks (Supplementary Fig. S2). Consequently, we argue that the activation rather than proliferation of γδT17 cells was inhibited by MA at concentrations without cytotoxicity in vitro.

### Screening of the signaling pathway accounting for madecassic acid inhibiting the activation of γδT17 cells

**Fig. 4:**
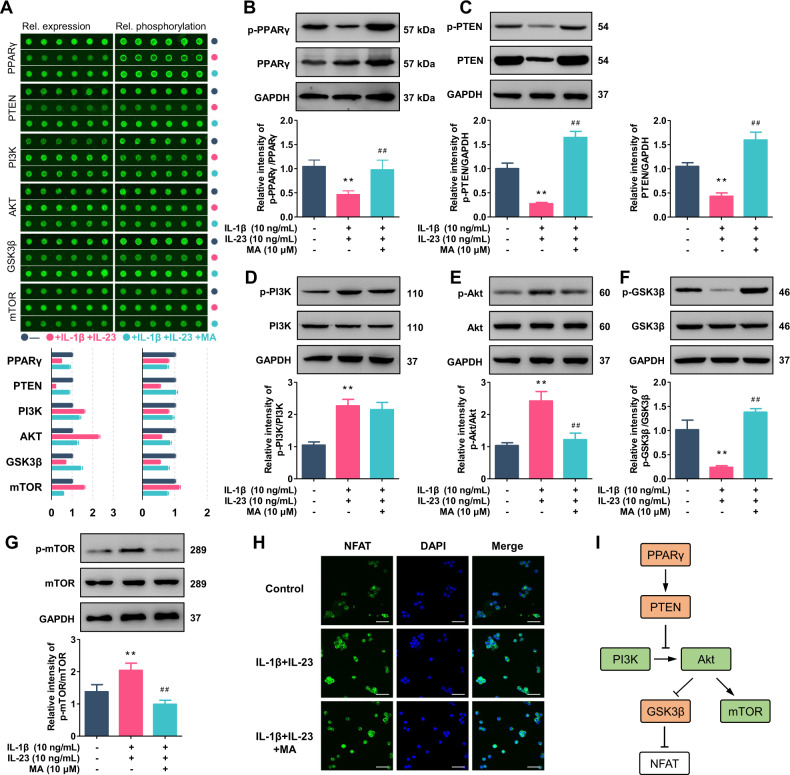
Screening of the signaling pathways accounting for madecassic acid inhibiting the activation of γδT17 cells. The γδT cells were stimulated with IL-1β (10 ng/mL) and IL-23 (10 ng/mL) for 72 h in the presence or absence of madecassic acid (MA, 10 μM). **a** The fluorescent images of the relative protein phosphorylation and expression as detected by the phospho explorer antibody array. Data are presented as fold change after normalization to untreated cells. **b**, **g** The expression and phosphorylation levels of PPARγ, PTEN, PI3K, AKT, GSK3β, and mTOR, as detected by western blot. **h** The localization of NFATc1 as visualized by immunofluorescence analysis (scale bar, 20 μm). **i** Schematic diagram of activated or deactivated proteins of the signaling pathway upon MA treatment (according to www.phosphosite.org). The data are expressed as means ± SEM from six technical replicates in microarray experiments or from three independent experiments. ^**^P < 0.01 versus the untreated group; ^##^*P* < 0.01 versus IL-1β and IL-23-stimulated group.

At present, the activation mechanism of γδT17 cells remains obscure. To explore the potential pathways by which MA inhibits the activation of γδT17 cells, an antibody microarray analysis was performed to identify the changes in protein expression and phosphorylation following MA treatment (Supplementary Fig. S3). In the activated γδT17 cells, MA (10 μM) significantly inhibited the phosphorylation of PPARγ (Fig. 4a, b), and reduced the expression and phosphorylation of PTEN (Fig. 4a, c). MA hardly affected the phosphorylation of PI3K (Fig. 4a, d), but suppressed the phosphorylations of Akt (Fig. 4a, e) and mTOR (Fig. 4a, g), and promoted the phosphorylation of GSK3β (Fig. 4a, f). Notably, the inactivation of mTOR by rapamycin was unable to inhibit the activation of γδT17 cells, and an overactivation of mTOR caused by the treatment of L-leucine did not affect the inhibitory effect of MA on the activation of γδT17 cells (Supplementary Fig. S4), suggesting that mTOR might be unlikely to be essential for the inhibition of MA on γδT17 cell activation. In addition, the promotion of the phosphorylation of GSK3β, an enhancer of nuclear export of NFAT (nuclear factor of activated T cells), suggests a possibility that MA can reduce the nuclear localization of NFATc1. Further immunofluorescence assay demonstrated that MA was indeed able to impede the nuclear localization of NFATc1 in the activated γδT17 cells (Fig. 4h). Moreover, FK506 (a NFAT inhibitor) could inhibit the activation of γδT17 cells and the expression of *Il17a* and *Rorc* (Supplementary Fig. S5). These findings suggest that inhibition of γδT17 cell activation by MA might involve the PPARγ–PTEN/Akt/GSK3β/NFAT pathway.

### Madecassic acid reduces the nuclear localization of NFATc1 in γδT17 cells through inhibition of Akt phosphorylation

**Fig. 5:**
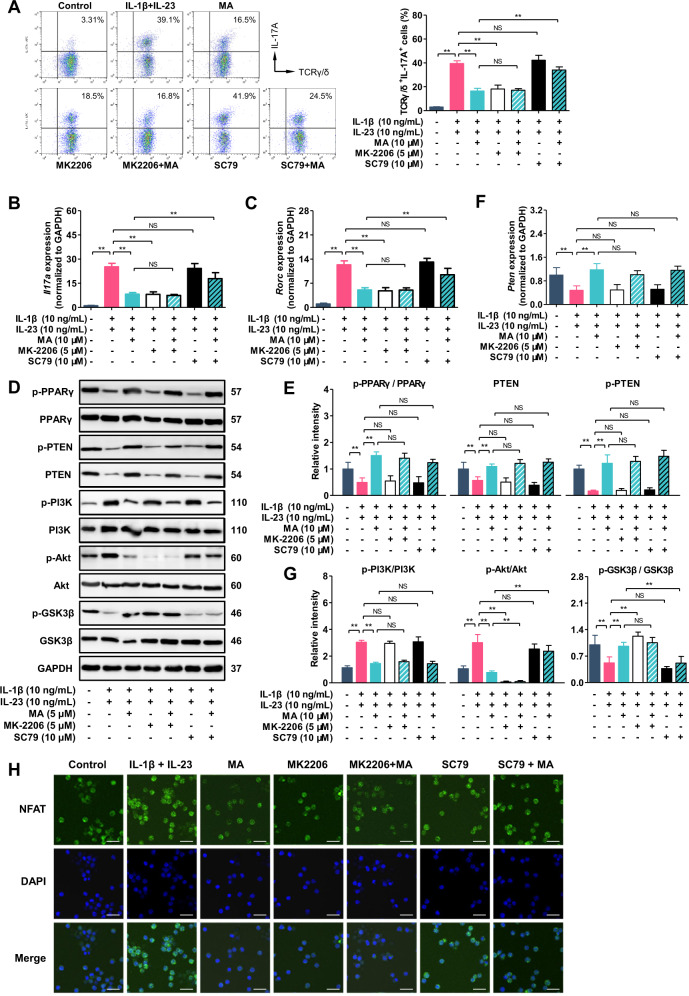
Madecassic acid reduces the nuclear translocation of NFATc1 in γδT17 cells through inhibiting AKT phosphorylation. The γδT cells were stimulated with IL-1β (10 ng/mL) and IL-23 (10 ng/mL) for 72 h in the presence or absence of madecassic acid (MA, 10 μM) or in combination with MK-2206 (5 μM) or SC-79 (10 μM). **a** The representative flow cytometry and percentages of IL-17A^+^ subpopulations present in the γδTCR^+^ T cells. **b**, **c** The expression levels of *Il17a* and *Rorc* as assessed by real-time PCR. **d**–**g** The expression and phosphorylation levels of PPARγ, PTEN, PI3K, AKT, GSK3β, and mTOR as detected by western blot. **f** The expression level of *Pten* as assessed by real-time PCR. **h** The localization of NFATc1 as visualized by immunofluorescence analysis (scale bar, 20 μm). The data are expressed as means ± SEM from three independent experiments. ^*^P < 0.05, ^**^P < 0.01 versus the indicated group.

In αβT cells, αβTCR signal-induced phosphorylation of Akt requires CD28 costimulation^23^. In contrast, in γδT cells, the γδTCR signal can stimulate Akt activation in the absence of costimulatory signals^24^. In order to recognize the role that Akt plays in the inhibition of MA on γδT17 cell activation, we used MK-2206 (a highly selective inhibitor of Akt) to inhibit the phosphorylation of Akt, and SC-79 (an activator of Akt phosphorylation) to restore Akt activation in γδT17 cells. It was shown that, similar to MA, MK-2206 could inhibit the activation of γδT17 cells and the expression of *Il17a* and *Rorc*. When MA was used in combination with SC-79, the inhibitory effect of MA on the activation of γδT17 cells nearly disappeared (Fig. 5a). Likewise, SC-79 almost abolished the inhibitory effect of MA on the expression of *Il17a* and *Rorc* (Fig. 5b, c). These results indicate that the inhibition of Akt phosphorylation by MA substantially contributes to the prevention of γδT17 cell activation.

In addition to mTOR that was excluded to participate in the inhibition of MA on γδT17 cell activation (Supplementary Fig. S4), GSK3β is another important downstream molecule of Akt, and phosphorylated GSK3β is capable of promoting NFAT translocation from the nucleus into the cytoplasm^25^. Our further studies showed that MK-2206 increased the phosphorylation of GSK3β, and reduced the nuclear localization of NFATc1 in γδT17 cells (Fig. 5d, g, h), but MK-2206 had no effect on the phosphorylation of PPARγ and PTEN (Fig. 5d–f). When used in combination with SC-79, MA failed to promote the phosphorylation of GSK3β (Fig. 5d, g) and prevent the nuclear localization of NFATc1 in γδT17 cells (Fig. 5h), but SC-79 did not alter the effect of MA on the phosphorylation of PPARγ and PTEN (Fig. 5d–f). These results suggest that MA promotes GSK3β activation and further reduces the nuclear localization of NFATc1 in γδT17 cells through inhibition of Akt phosphorylation.

### Madecassic acid inhibits the PTEN/PI3K–Akt/GSK3β/NFAT signal pathway in γδT17 cells by activating PPARγ

**Fig. 6:**
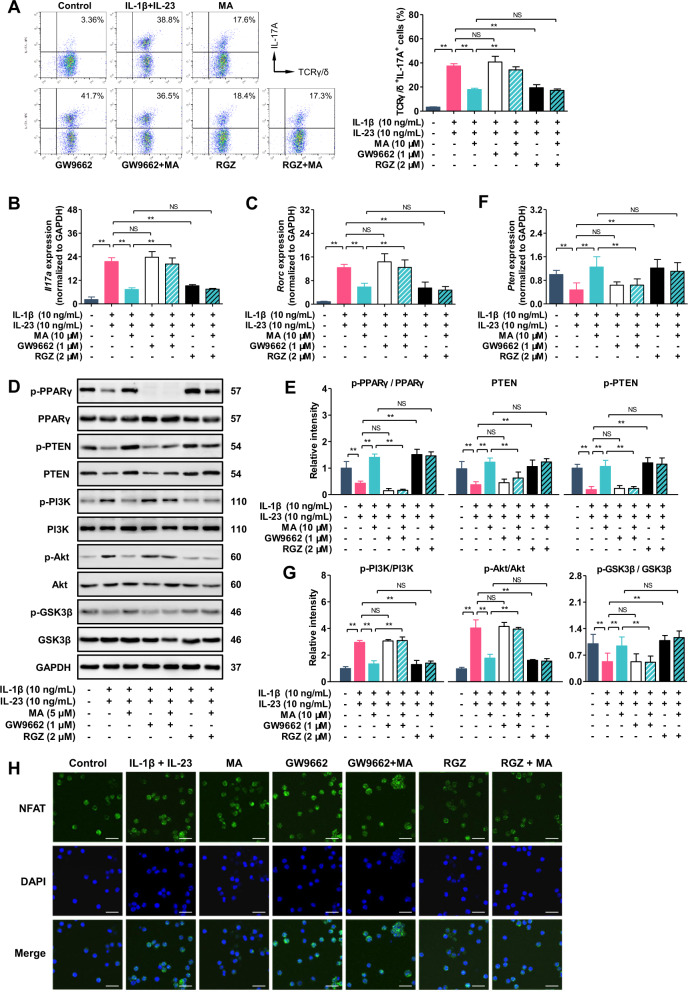
Madecassic acid inhibits the PI3K–Akt/NFAT signal pathway in γδT17 cells by activating PPARγ. The γδT cells were stimulated with IL-1β (10 ng/mL) and IL-23 (10 ng/mL) for 72 h in the presence or absence of madecassic acid (MA, 10 μM) or in combination with GW9662 (1 μM) or rosiglitazone (RGZ, 2 μM). **a** The representative flow cytometry and percentages of IL-17A^+^ subpopulations present in the γδTCR^+^ T cells. **b**, **c** The expression levels of *Il17a* and *Rorc* as assessed by real-time PCR. **d**–**g** The expression and phosphorylation levels of PPARγ, PTEN, PI3K, AKT, GSK3β, and mTOR, as detected by western blot. **f** The expression level of *Pten* as assessed by real-time PCR. **h** The localization of NFATc1, as visualized by immunofluorescence analysis (scale bar, 20 μm). The data are expressed as means ± SEM from three independent experiments. ^*^P < 0.05, ^**^P < 0.01 versus the indicated group.

Our previous studies demonstrated that MA is a PPARγ agonist^16^. The activation of PPARγ could increase PTEN expression, and inhibit Akt phosphorylation^26^. A recent report indicated that the activation of γδT17 cells could be promoted by SF1670 (an inhibitor of PTEN)^27^. To further verify the key role of PPARγ in the inhibitory effect of MA on γδT17 cell activation, we used rosiglitazone (RGZ, a selective PPARγ agonist) to activate PPARγ and GW9662 (a selective and irreversible PPARγ antagonist) to inhibit the activation of PPARγ in γδT17 cells. It was shown that RGZ inhibited the activation of γδT17 cells and the expression of *Il17a* and *Rorc* (Fig. 6a–c). In addition, in combination with GW9662, the inhibitory effect of MA on the activation of γδT17 cells nearly disappeared (Fig. 6a). Likewise, GW9662 almost abolished the inhibition of MA on the expression of *Il17a* and *Rorc* (Fig. 6b, c). Therefore, we argued that MA-mediated PPARγ activation contributed to the inhibition of γδT17 cell activation. As expected, RGZ increased the phosphorylation of PPARγ in γδT17 cells (Fig. 6d, e). Further experiments showed that RGZ increased the expression and phosphorylation of PTEN (Fig. 6d–f), and promoted the phosphorylation of Akt (Fig. 6d, g). Correspondingly, the phosphorylation of GSK3β was promoted (Fig. 6d, g), and the nuclear localization of NFATc1 was reduced by RGZ (Fig. 6h). In addition, the promotion effect of MA on the expression and phosphorylation of PTEN (Fig. 6d–f) and the inhibitory effect of MA on phosphorylation of Akt were restored (Fig. 6d, g) when γδT17 cells were treated with GW9662 and MA. In contrast, the promotion effect of MA on the phosphorylation of GSK3β nearly disappeared (Fig. 6d, g) and the nuclear localization of NFATc1 in γδT17 cells was restored (Fig. 6h) when γδT17 cells were treated with GW9662 and MA. These results suggest that MA inhibits the PTEN/Akt/GSK3β/NFAT signal pathway in γδT17 cells by activating PPARγ.

### Madecassic acid alleviates colitis through inhibiting the activation of γδT17 cells dependent on PPARγ

**Fig. 7:**
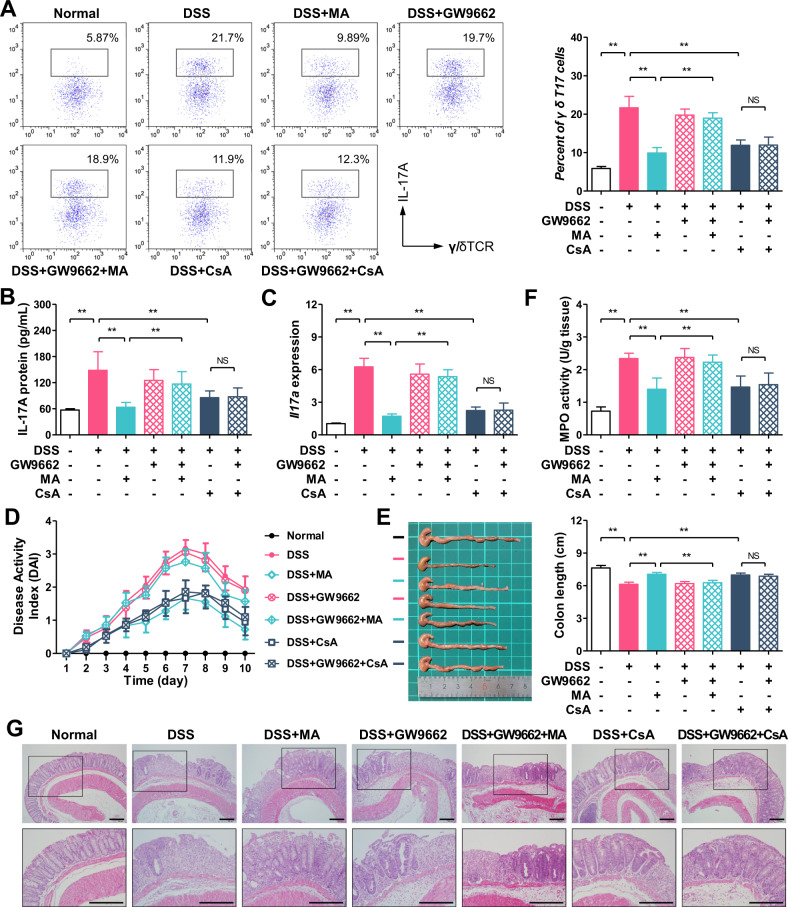
Madecassic acid alleviates colitis through inhibiting the activation of γδT17 cells dependent on PPARγ. Colitis was induced in female C57BL/6 mice by drinking with 2.5% dextran sulfate sodium (DSS) for 7 days, and followed by normal drinking water for 3 days. Madecassic acid (MA, 25 mg/kg), cyclosporin A (CsA, 25 mg/kg), or GW9662 (1 mg/kg) were orally administered for 10 days consecutively. **a** The representative flow cytometry and percentages of IL-17A^+^ subpopulations present in the γδTCR^+^ T cells from the colon tissues of mice. **b** The levels of IL-17A as assessed by enzyme-linked immunosorbent assay. **c** The expression level of *Il17a* as assessed by real-time PCR. **d** Disease activity index (DAI). **e** Colon length. **f** Myeloperoxidase (MPO) activity. **g** Hematoxylin and eosin (H&E) staining (scale bar, 100 μm). The data are expressed as means ± SEM of six mice per group. ^*^P < 0.05, ^**^P < 0.01 versus the indicated group.

To further ascertain the causal link between PPARγ activation, inhibition of γδT17 activation, and anti-colitis effects of MA, MA was orally administered in combination with GW9662 to colitis mice. The results showed that GW9662 counteracted the inhibitory effect of MA on the activation of γδT17 cells in the colons of colitis mice, but it did not affect the inhibitory effect of CsA (Fig. 7a). A plausible explanation for this phenomenon is that CsA prevents nuclear import of NFAT by blocking calcineurin activity^28^; however, MA reduces the nuclear localization through the PPARγ–PTEN/Akt/GSK3β pathway. As expected, MA inhibited the expression of *Il17a* and IL-17A in the colons, and these effects were counteracted by GW9662, but the reduction of IL-17A expression caused by CsA was not markedly weakened by GW9662 (Fig. 7b, c). These results indicate that MA, different from CsA, inhibits the activation of γδT17 cells by activating PPARγ. In addition, GW9662 markedly weakened the anti-colitis effect of MA as evidenced by higher DAI (Fig. 7d), shorter colon length (Fig. 7e), higher MPO activity (Fig. 7f), and more serious pathological lesion (Fig. 7g) in the colon tissues. These findings reveal that MA alleviates colitis through inhibiting the activation of γδT17 cells dependent on PPARγ.

## Discussion

IL-17, an acknowledged pro-inflammatory cytokine, plays an important role in UC. Clinical studies showed that IL-17 was highly expressed in intestinal mucosal tissues of patients with UC^29,30^. In animal studies, DSS-induced colitis was IL-17-dependent^31,32^. These findings indicated that IL-17 is associated with the development and progression of UC, and suggested that IL-17 is an attractive therapeutic target. For many years, researchers have been exploring the new IL-17-based treatment schemes^33–35^, although the results of clinical trials with anti-IL-17 antibodies in inflammatory bowel disease (IBD) were discouraging^36^. Our previous study demonstrated that orally administered MA could obviously attenuate colitis by downregulating the expression of IL-17 in colon tissue of colitis mice^16^, but the IL-17-producing effector cells remain undefined.

It is generally accepted that IL-17 is produced mainly by Th17-type lymphocytes in colon tissue of colitis^37,38^. Some studies have also pointed out that IL-17 is also largely secreted by γδT cells^39,40^ or CD8^+^ T cells^41,42^. This study found that MA could reduce the level of IL-17 and the number of γδT17 cells in colon tissues of colitis mice, and attenuate colitis in mice. In the absence of suitable genetic tools for the ablation of IL-17 specifically in γδT-cell lineage, some conclusions, based on associations of phenotypes of mice lacking γδT cells or IL-17, are unconvincing^43^. The contribution of γδT17 cells to diseases can be verified by adoptive transfer of γδT17 cells^43^. This strategy has been employed in colitis mouse models^44^. In this study, the anti-colitis effect of MA was counteracted by adoptive transfer of γδT17 cells, which suggested that γδT17 was the main target cell of MA ameliorating colitis and reducing the expression of IL-17 in colon tissues of colitis mice.

Unlike conventional type-17 αβT effector cells, γδT17 cells acquire their effector potential in the thymus before their distribution to the periphery^21–23^. These preprogrammed effector cells can rapidly switch from rest to activated state and secrete a large amount of cytokines during the immune response^45–47^. Recent researches have shown that γδT17 cells are deeply involved in multiple inflammation and autoimmune diseases, such as colitis^17^, psoriasis^48^, asthma^49^, diabetes^50^, encephalomyelitis^51^, and so on. The IL-17 produced from γδT cells can independently promote inflammation development^52^. On the other hand, γδT17 cells can also amplify Th17 responses in inflammation^53,54^. In this study, the number of activated γδT17 cells in colon tissues of colitis mice was decreased by orally administering MA. Moreover, the activation of γδT17 cells was inhibited by MA at concentrations without cytotoxicity in vitro.

It is reported that TCR signaling is a major determinant of the function of effector γδT cells^55^. However, little is known about the mechanism of γδTCR to drive IL-17 secretion in γδT cells. A study identified that γδ17 cells are absent in mice with defective Zap-70 function^56^. However, another recent study indicated the Syk signaling pathway was one means to control the production of IL-17 in γδT cells^27^. In fact, stimulation with IL-1β and IL-23 is sufficient to trigger abundant secretion of IL-17 by γδT cells in vitro, even in the absence of TCR stimulation^43^. IRF4 cooperates with STAT3 to regulate gene expression, and links IL-1R and IL-23R signaling pathways to IL-17 production^3^. STAT3 activation is crucial for RORγt expression in Th17 cells and also important for IL-17 production in γδT cells^57,58^. Furthermore, both classical and noncanonical NF-κB signaling pathways are important for γδT17. RelA or RelB conditional deficiency leads to reduction of γδT17 cells through reducing *Il17a* and *Rorc* expression at the transcriptional level, and p52, not p50, was also required for IL-17 production^59^. NF-κB-inducing kinase, which is required for noncanonical NF-κB signaling, is essential for IL-17 production as NF-κB-inducing kinase depletion led to impairing *Rorc* and *Sox13* expression^60^. Notch signaling and its downstream target Hes1, one of the basic helix–loop–helix proteins, are indispensable for the IL-17-producing function of mature γδT cells in the periphery^61^. In this study, we found that MA could inhibit the activation of γδT17 cells through the PPARγ–PTEN/Akt/GSK3β/NFAT pathway.

The nuclear receptor PPARγ, a ligand-activated transcription factor that plays an important role in the control of gene expression linked to a variety of physiological processes, is highly expressed in the colon^62,63^. Many preclinical researchers have pointed out that the activation of PPARγ plays a protective role in mice with colitis^64–66^. Many studies have shown that various compounds, acting as the agonists of PPARγ (such as rosiglitazone, troglitazone, and thiazolidinediones), have immunoprotective roles in experimental colitis^67–69^. In our previous study, MA has shown to induce Th17 toward Treg cells in a PPARγ-dependent manner and ameliorate experimental colitis by activating PPARγ^16^. In this study, MA can ameliorate experimental colitis through inhibiting the activation of γδT17 cells in a PPARγ-dependent manner.

In conclusion, γδT17 cells play an important role in the anti-colitis effect of MA. MA is able to inhibit the activation of γδT17 cells through PPARγ–PTEN/Akt/GSK3β/NFAT pathway, reduce the level of IL-17 in colon tissues of colitis mice, and ameliorate DSS-induced colitis in mice. MA and other PPARγ agonists are promising for the prevention and treatment of γδT17-related IBD or other diseases.

## Materials and methods

### Media and reagents

Madecassic acid (CAS no. 18449-41-7) with a purity of 98.5% was purchased from Jiangsu Yongjian Pharmaceutical Technology Co., Ltd (Taizhou, China). DSS (MW: 36–50 kDa) was purchased from MP Biomedicals Inc. (Irvine, CA, USA). Mouse IL-17 ELISA kit was purchased from Lianke Biotech Co., Ltd (Hangzhou, China). MPO activity assay kit was obtained from Nanjing Jiancheng Bioengineering Institute (Nanjing, China). FITC-anti-mouse CD3, PE-anti-mouse TCR γ/δ, PE-anti-mouse CD4, PE-anti-mouse CD8, and APC-anti-mouse IL-17A were purchased from eBioscience, Inc. (San Diego, CA, USA). Murine IL-1β and IL-23 were purchased from Sino Biological Inc. (Beijing, China). Mouse TCRγ/δ T Cell Isolation Kit was purchased from Miltenyi Biotec Inc. (Bergisch Gladbach, Germany). TRIzol reagent was purchased from SunShine Biotechnology Co., Ltd (Nanjing, China). PPARγ, phosphorylated (p)-PPARγ, PTEN, p-PTEN, PI3K, p-PI3K, Akt, p-Akt, GSK3β, p-GSK3β, mTOR, p-mTOR, NFATc1, and glyceraldehyde 3-phosphate dehydrogenase (GAPDH) mAb were purchased from HuaBio Inc. (Hangzhou, China). Cyclosporin A, GW9662, MK-2206, and rapamycin were obtained from CSNpharm (Chicago, USA). Rosiglitazone, FK506, LY294002, and SC-97 were purchased from Selleck (Houston, USA).

### Animals

Female C57BL/6 mice (20 ± 2 g) were obtained from the Comparative Medicine Center of Yangzhou University (Yangzhou, China). Mice were maintained in the animal center of China Pharmaceutical University, a specific pathogen-free environment, with light:dark (12 h:12 h) condition and fed with commercial diet and water ad libitum. All animal experiments were approved by the animal ethics committee of the China Pharmaceutical University, and were performed in accordance with the Guide for the Care and Use of Laboratory Animals.

### DSS-induced colitis in mice

All mice were assigned to different groups according to a randomized block experimental design. Experimental colitis was induced in mice by freely drinking of 2.5% DSS for 7 days followed by normal drinking water for 3 days. Madecassic acid (25 mg/kg) and cyclosporin A (25 mg/kg) were orally gavaged daily for 10 days, and carboxyl methyl cellulose sodium (CMC-Na, 0.5%) was used as the vehicle control. Body weight, diarrhea, and rectal bleeding were measured every day. The DAI was accounted by the mean value of the following three elements. Briefly, (a) body weight loss (0 = none, 1 = 1–5%, 2 = 5–10%, 3 = 10–15%, and 4 = over 15%), (b) stool consistency (0 = normal, 2 = loose stools, and 4 = diarrhea), and (c) gross bleeding (0 = normal, 2 = hemoccult, and 4 = gross bleeding).

On day 10, mice were sacrificed, the colon length was measured, and then the colon was fixed in 4% PBS-buffered formaldehyde as a roll and embedded in paraffin. The 5-mm tissue sections were stained with hematoxylin and eosin (H&E). Histological scores were graded as follows: (a) severity of inflammation: 0 = none, 1 = slight, 2 = moderate, and 3 = severe, (b) sites of inflammation: 0 = none, 1 = mucosa, 2 = mucosa and submucosa, and 3 = transmural, and (c) lesions of crypt: 0 = none, 1 = basal 1/3 damaged, 2 = basal 2/3 damaged, 3 = only surface epithelium intact, and 4 = entire crypt and epithelium lost. The histological score was assessed by the average of the three evaluations with a maximal score of 10.

### Real-time quantitative PCR assay

The Trizol reagent was employed to extract total mRNA from colonic homogenates or cultured cells. The mRNA was reverse-transcribed using 5×All-In-One RT MasterMix kit according to the manufacturer’s instructions. RT-qPCR analysis was performed with EvaGreen 2×qPCR MasterMix kit on a Bio-Rad iQ Real Time PCR system (Hercules, CA, USA). The RT-qPCR conditions were activation at 95 °C for 10 min, followed by 35 cycles of amplification with denaturation at 95 °C for 15 s, and then annealing and extension at 60 °C for 60 s. The primer sequences used are listed in Table 1. GAPDH was used as an internal control. The expression of each gene was normalized to the corresponding GAPDH threshold cycle (Ct) values by the 2^−ΔΔCt^ method.

**Table 1:** Oligonucleotide sequences of quantitative real-time PCR.

Gene	Primer	Sequences (5′-3′)
IL-17A	Forward	CACCGCAATGAAGACCCTGA
	Reverse	TTCCCTCCGCATTGACACAG
IL-17F	Forward	ACGTGAATTCCAGAACCGCT
	Reverse	TTGGAGATCGGGCTTCACAC
IL-21	Forward	ACTCAGTTCTGGTGGCATGG
	Reverse	TGAATCATCTTTTGAAGGAGCCA
IL-22	Forward	TTGTGCGATCTCTGATGGCT
	Reverse	GAAGGCAGGAAGGAGCAGTT
RORγt	Forward	ACAGCCACTGCATTCCCAGTTT
	Reverse	TCTCGGAAGGACTTGCAGACAT
Sox4	Forward	GAACGCCTTTATGGTGTGGT
	Reverse	GAACGGAATCTTGTCGCTGT
Sox13	Forward	GAACAGCAGCCACATCAAGA
	Reverse	TGCTGATGCTGGAGTTATGC
PLZF	Forward	AGAAGACCCACACCTCACAAA
	Reverse	TCTGGTCATTCTGGCAGAGC
IL-1R	Forward	AGCCAGGAGACAAAGATGGC
	Reverse	TCGGGGTCTGAACACAACTG
IL-23R	Forward	ACACCGGACAAACCAAAGAC
	Reverse	ATGCCGGGAGCTATCTTTCT
CCR2	Forward	TGCGACTTCAACAGCAACTC
	Reverse	ATGTAGGCAATGAGGTCCAC
CCR6	Forward	CAGTCCAACCTTGGGATGCT
	Reverse	GTGCCCTTAGTCTTCAGCGT
PTEN	Forward	TAGCCCACGTCGTAGCAAAC
	Reverse	ACCCTGAGCCATAATCCCCT
GAPDH	Forward	AGAAGGCTGGGGCTCATTTG
	Reverse	AGGGGCCATCCACAGTCTTC

### Enzyme-linked immunosorbent assay

Colonic tissues were homogenated, and the supernatants were collected for the detection of IL-17A level by enzyme-linked immunosorbent assay kits according to the manufacturer’s instructions.

### Extracellular staining and flow cytometry

For extracellular staining of immune markers, we prepared lamina propria mononuclear cell suspensions by mechanic dispersion and enzymatic digestion of colon tissues. Lamina propria mononuclear cells and γδT cells were resuspended in RPMI-1640 supplemented with 10% fetal bovine serum labeled with FITC-anti-mouse CD3, and PE-anti-mouse TCR γ/δ or PE-anti-mouse CD4 or PE-anti-mouse CD8 at 4 °C for 30 min. After fixation and permeabilization, the cells were labeled with APC-anti-mouse IL-17A at 4 °C for 30 min. All flow cytometric measurements were conducted on FACS Calibur (BD Biosciences, USA). The results were analyzed by Flowjo 10.0.1 (Flowjo LLC, USA).

### Western blot analysis

Cell and tissue lysate homogenates were prepared by NP-40 buffer. Following the mixture with SDS-PAGE protein sample buffer, proteins were divided by SDS-PAGE (10%). Subsequently, the protein was transferred to nitrocellulose filter membranes. After that, the nitrocellulose filter membranes were transiently blocked by 5% defatted milk for about 2 h at room temperature, and then incubated with primary antibodies overnight at 4 °C. After washing with TBST, the membranes were incubated with the appropriate secondary antibody for 2 h at room temperature. After washing, the hybridized bands were obtained with the help of Odyssey Infrared Imaging System.

### Immunofluorescence staining

Cells were fixed with 4% paraformaldehyde for 15 min and permeabilized by 0.1% Triton X-100 for 15 min. After blocking in 4% bovine serum albumin for 2 h, the cells were incubated with primary antibodies at 4 °C overnight, and next with fluorophore rhodamine- or FITC-conjugated IgG for 2 h, and nuclei were counterstained with DAPI for 10 min in the dark, followed by three washes with PBST. Anti-fluorescence quenching agent was added and photographed using a fluorescence microscope.

### Microarray analysis

The phospho explorer antibody microarray, which was designed and manufactured by Full Moon Biosystems, Inc. (Sunnyvale, CA), contains 304 antibodies. Each of the antibodies has six replicates that are printed on coated glass microscope slides, along with multiple positive and negative controls. The antibody array experiment was performed by Huaying Biotechnology (Shanghai, China), according to their established protocol. Briefly, the cell lysates, which were obtained from γδT cells treated with IL-1β and IL-23 or madecassic acid, were biotinylated with an Antibody Array Assay Kit. The antibody microarray slides were first blocked in a blocking solution. Then the biotin-labeled cell lysate was placed on preblocked microarray slides. After washing, bound biotinylated proteins were detected using Cy3-conjugated streptavidin. The slides were scanned on a GenePix 4000 scanner, and the images were analyzed with GenePix Pro 6.0 (Molecular Devices, Sunnyvale, CA). Where indicated, protein phosphorylation data were confirmed by western blotting.

### Sorting and purification of γδT cells

Spleens were harvested and cut into small fragments mechanically. Cut fragments were crushed between nylon mesh (300 meshes). Then red blood cells were depleted by red blood cell lysis buffer, and the pellet was washed in phosphate-buffered saline (PBS).

The γδT cells were isolated from single-spleen cell suspensions using a mouse γδTCR^+^ T Cell Isolation Kit (MACS) according to the manufacturer’s instruction. Briefly, the isolation of mouse γδT cells is performed in a two-step procedure. To pre-enrich the target cells, non-T cells are magnetically labeled with a cocktail of CD45R and CD11b antibodies conjugated to MicroBeads. Concomitantly, γ/δTCR^+^ T cells are labeled with Anti-γ/δTCR-Biotin included in the cocktail. The magnetically labeled, unwanted cells are subsequently depleted by separation over a MACS Column. In the second step, γ/δTCR^+^ T cells are indirectly magnetically labeled with Anti-Biotin MicroBeads and isolated by positive selection from the pre-enriched T-cell fraction. The magnetically labeled γ/δTCR^+^ T cells are retained on the column and eluted after removal of the column from the magnetic field. Then, the absolute cell numbers were calculated.

The purified γδT cells were incubated in RPMI-1640 medium supplemented with 10% fetal bovine serum (FBS), 1% penicillin–streptomycin, 5 μM zoledronate, and 100 IU/mL IL-2 at 37 °C, 5% CO_2_ for 2 weeks, which was refreshed twice weekly. γδT-cell purity was determined by flow cytometry with purity over 90%, which can be used for further experiments.

### Adoptive transfer of γδT cells

γδT cells were cultured with IL-1β (10 ng/mL) and IL-23 (10 ng/mL) for 72 h. Then, the cells were adoptively transferred into the recipient mice by intravenous injection at the 3rd and 7th day (10^5^ cells/mouse in 200 μL of PBS). The same volume of PBS was used as the control.

### Assessment of colonic MPO activity

The activity of MPO is a mark of neutrophil infiltration, which reflects the number and distribution of neutrophils in the tissues. The colon tissues were precisely weighed and homogenized with PBS (1:9, w/v). The supernatants were collected. The MPO activity was measured according to the manufacturer’s instructions from JianCheng Bioengineering Institute (Nanjing, China).

### Histological evaluation of the colon

To assess the inflammatory level, murine colonic tissue of the colons was collected and fixed in 10% neutral buffered formalin at room temperature, embedded in paraffin, and sliced. For histological evaluation, 5-μm-thick tissue sections were stained with H&E and examined under the light microscope. The investigators were blinded to the group allocation when assessing the outcome.

### Statistical analysis

The data are expressed as the mean ± SEM. Statistically significant differences between the groups were determined by one-way analysis of variance (ANOVA) followed by Tukey’s multiple- comparison test. At least three independent replicates of each experiment were conducted. Differences were considered statistically significant at ^*^P < 0.05 or ^**^P < 0.01.

## Supplementary information

Figure S1

Figure S2

Figure S3

Figure S4

Figure S5
