# Supplementary material for: Inhibition of the activation of γδT17 cells through PPARγ–PTEN/Akt/GSK3β/NFAT pathway contributes to the anti-colitis effect of madecassic acid
Source: Cell Death Dis. 2020 Sep 14;11(9):752. doi: 10.1038/s41419-020-02969-x (PMC7490397; doi:10.1038/s41419-020-02969-x)
Supplement: Supplementary file 1 — Figure S1 [file 41419_2020_2969_MOESM1_ESM.docx]

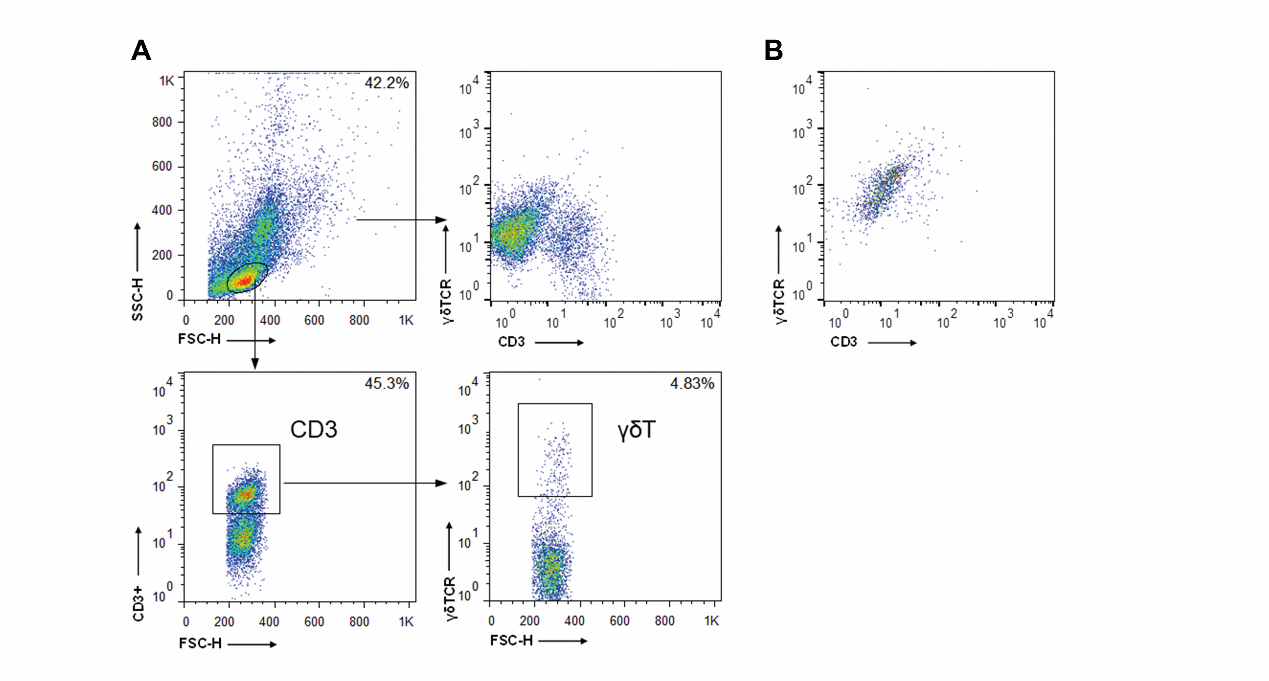


**Figure S1 Identification and sorting of γδT cells.** (A) Gating strategy to detect γδT cells. Selection of lymphocytes → Selection of CD3^+^ lymphocytes → Selection of γδTCR^+^ T cells. (B) γδT cells isolated from the mouse spleens.
