# Supplementary material for: Inhibition of the activation of γδT17 cells through PPARγ–PTEN/Akt/GSK3β/NFAT pathway contributes to the anti-colitis effect of madecassic acid
Source: Cell Death Dis. 2020 Sep 14;11(9):752. doi: 10.1038/s41419-020-02969-x (PMC7490397; doi:10.1038/s41419-020-02969-x)
Supplement: Supplementary file 2 — Figure S2 [file 41419_2020_2969_MOESM2_ESM.docx]

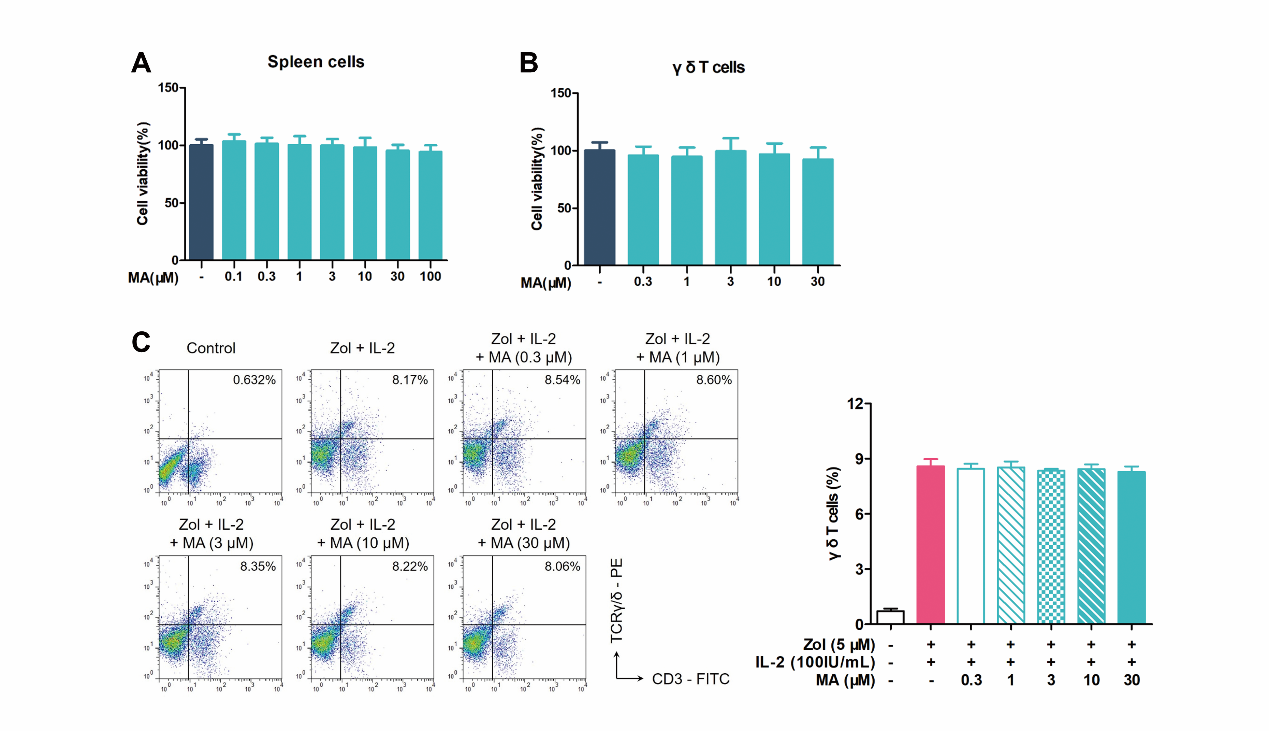


**Figure S2** **Effect of madecassic acid on the viability and expansion of γδT cells isolated from mouse spleens.** (A&B) Cell viability as detected using MTT assay. The spleen cells or γδT cells were exposed to different concentrations of madecassic acid (MA) for 72 h, and the fresh medium containing 0.1% DMSO was used as the negative control. (C) The number of γδT cells as detected by flow cytometry. The γδT cells were stimulated with zoledronate (Zol, 5 μM) and IL-2 (100 IU/mL), and exposed to different concentrations of MA for 2 weeks. The data are expressed as means ± SEM from 3 independent experiments.
