# Supplementary material for: Inhibition of the activation of γδT17 cells through PPARγ–PTEN/Akt/GSK3β/NFAT pathway contributes to the anti-colitis effect of madecassic acid
Source: Cell Death Dis. 2020 Sep 14;11(9):752. doi: 10.1038/s41419-020-02969-x (PMC7490397; doi:10.1038/s41419-020-02969-x)
Supplement: Supplementary file 3 — Figure S3 [file 41419_2020_2969_MOESM3_ESM.docx]

**Figure S3 The full fluorescent images of relative protein expression and phosphorylation in γδT17 cells.** The γδT cells were stimulated with IL-1β (10 ng/mL) and IL-23 (10 ng/mL) for 72 h in the presence of madecassic acid (MA, 10 μM). Then the γδT cell lysates were biotinylated with an Antibody Array Assay Kit, and the biotin-labeled cell lysates were placed on preblocked microarray slides. The bound biotinylated proteins were detected by using Cy3-conjugated streptavidin. The slides were scanned on a GenePix 4000 scanner. (A) The sites of 304 antibodies on microarray. The relevant proteins and phosphorylations were highlighted. (B) The full fluorescent images as detected by the phospho explorer antibody microarray.

**A**

| Position Marker | Empty | Empty | Tubulin-b | GAPDH | GSK3 alpha/beta (Phospho-Tyr216/279) | GSK3 beta | GSK3 beta (Phospho-Ser9) | c-Jun | c-Jun (Phospho-Ser73) | Elk1 | Elk1 (Phospho-Ser383) | PDK | PDK1 (Phospho-Ser241) | Raf1 | Raf1 (Phospho-Ser259) | NFkB-p100/p52 | NFkB-p100/p52 (Phospho-Ser865) | NFkB-p105/p50 | NFkB-p105/p50 (Phospho-Ser337) | JunB | JunB (Phospho-Ser259) | ATF2 | ATF2 (Phospho-Ser62/44) | STAT1 | STAT1 (Phospho-Tyr701) | STAT3 | STAT3 (Phospho-Tyr705) | STAT4 | STAT4 (Phospho-Tyr693) | STAT5A | STAT5A (Phospho-Tyr694) |
| --- | --- | --- | --- | --- | --- | --- | --- | --- | --- | --- | --- | --- | --- | --- | --- | --- | --- | --- | --- | --- | --- | --- | --- | --- | --- | --- | --- | --- | --- | --- | --- |
| STAT6 | STAT6 (Phospho-Thr645) | CREB | CREB (Phospho-Ser133) | AKT | AKT1 | AKT1 (Phospho-Ser473) | AKT1 (Phospho-Thr308) | PTEN | PTEN (Phospho-Ser380/Thr392/Thr383) | P73 | P73 (Phospho-Tyr99) | Integrin beta-3 | Integrin beta-3 (Phospho-Tyr773) | Chk2 | Chk2 (Phospho-Thr68) | BCL-2 (Phospho-Ser70) | BCL-XL | BCL-XL (Phospho-Ser62) | BAD (Phospho-Ser136) | HER2 | HER2 (Phospho-Tyr1221/Tyr1222) | HER2 (Phospho-Tyr1248) | VEGFR2 | VEGFR2 (Phospho-Tyr1175) | VEGFR2 (Phospho-Tyr1214) | IGF1R | IGF1R (Phospho-Tyr1165/1166) | Src | Src (Phospho-Tyr418) | p53 | p53 (Phospho-Ser9) |
| p53 (Phospho-Ser15) | p53 (Phospho-Ser315) | Tau | Tau (Phospho-Ser396) | Tau (Phospho-Ser235) | Catenin beta | Catenin beta (Phospho-Thr41/Ser45) | CDC25C | CDC25C (Phospho-Ser216) | AKT2 | AKT2 (Phospho-Ser474) | c-Abl | c-Abl (Phospho-Tyr412) | Rb | Rb (Phospho-Ser608) | Rb (Phospho-Ser807) | IRS-1 | IRS-1 (Phospho-Ser312) | LCK | LCK (Phospho-Tyr393) | JAK1 | JAK1 (Phospho-Tyr1022) | JAK2 | JAK2 (Phospho-Tyr221) | JAK2 (Phospho-Tyr1007) | IkB-alpha | IkB-alpha (Phospho-Ser32/36) | FKHRL1/FOXO3A | FKHRL1/FOXO3A (Phospho-Ser253) | Zap-70 | Zap-70 (Phospho-Tyr319) | HDAC2 |
| HDAC2 (Phospho-Ser394) | MEK1 | MEK1 (Phospho-Ser221) | MEK1 (Phospho-Ser217) | MKK4/SEK1 | MKK4/SEK1 (Phospho-Thr261) | ASK1 | ASK1 (Phospho-Ser966) | HSP27 | HSP27 (Phospho-Ser15) | HSP27 (Phospho-Ser82) | MSK1 | MSK1 (Phospho-Ser376) | p21Cip1 | p21Cip1 (Phospho-Thr145) | VASP | VASP (Phospho-Ser157) | NFkB-p65 | NFkB-p65 (Phospho-Ser529) | NFkB-p65 (Phospho-Ser311) | EGFR | EGFR (Phospho-Tyr1172) | EGFR (Phospho-Tyr869) | mTOR | mTOR (Phospho-Ser2448) | mTOR (Phospho-Ser2481) | 4E-BP1 | 4E-BP1 (Phospho-Thr45) | Met | Met (Phospho-Tyr1234) | elF4E | elF4E (Phospho-Ser209) |
| BRCA1 | BRCA1 (Phospho-Ser1423) | CDK1/CDC2 | CDK1/CDC2 (Phospho-Tyr15) | p44/42 MAP Kinase | p44/42 MAP Kinase (Phospho-Tyr204) | SAPK/JNK | SAPK/JNK (Phospho-Tyr185) | P38 MAPK | P38 MAPK (Phospho-Thr180/Tyr182) | RelB | RelB (Phospho-Ser552) | DARPP-32 | DARPP-32 (Phospho-Thr34) | eIF2A | eIF2A (Phospho-Ser51) | P70S6K | P70S6K (Phospho-Ser424) | P70S6K (Phospho-Thr421) | Synuclein alpha | Synuclein alpha (Phospho-Tyr136) | CaMK2A | CaMK2A (Phospho-Thr286) | CPI17 alpha | CPI17 alpha (Phospho-Thr38) | Gab1 | Gab1 (Phospho-Tyr627) | NMDAR1 (Phospho-Ser897) | NMDAR2A/B (Phospho-Tyr1246/1252) | PKC delta | PKC delta (Phospho-Ser645) | PKC theta |
| PKC theta (Phospho-Thr538) | HSP90B | HSP90B (Phospho-Ser226) | IkB-epsilon | IkB-epsilon (Phospho-Ser22) | Elk1 | Myc (Phospho-Ser62) | Shc | Shc (Phospho-Tyr349) | SHP-2 | SHP-2 (Phospho-Tyr542) | Trk B | Trk B (Phospho-Tyr515) | CrkII | CrkII (Phospho-Tyr221) | eEF2K | eEF2K (Phospho-Ser366) | STAT5B | STAT5B (Phospho-Ser731) | SYK | SYK (Phospho-Tyr348) | ACC1 | ACC1 (Phospho-Ser80) | Caspase 8 | Caspase 8 | Caspase 8 (Phospho-Ser347) | EEF2 | EEF2 (Phospho-Thr56) | SRF | SRF (Phospho-Ser99) | STAT2 | STAT2 (Phospho-Tyr690) |
| AMPKbeta1 | AMPKbeta1 (Phospho-Ser182) | Cyclin D1 | Cyclin D1 (Phospho-Thr286) | IKK alpha/beta | IKK alpha/beta (Phospho-Ser180/181) | IKK-beta | IKK-beta (Phospho-Tyr188) | IKK-beta (Phospho-Tyr199) | IKK-gamma | IKK-gamma (Phospho-Ser31) | AKT1S1 | AKT1S1 (Phospho-Thr246) | BID | BID (Phospho-Ser78) | FOXO1/3/4-pan | FOXO1/3/4-pan (Phospho-Thr24/32) | GRK2 | GRK2 (Phospho-Ser29) | MDM2 | MDM2 (Phospho-Ser166) | P90RSK | P90RSK (Phospho-Thr359/Ser363) | PP2A-alpha | PP2A-alpha (Phospho-Tyr307) | Tuberin/TSC2 | Tuberin/TSC2 (Phospho-Thr1462) | Cyclin E1 | Cyclin E1 (Phospho-Thr77) | LKB1 | LKB1 (Phospho-Thr189) | Ras-GRF1 |
| Ras-GRF1 (Phospho-Ser916) | 14-3-3 theta/tau (Phospho-Ser232) | 14-3-3 zeta/delta | Actin Pan | Actin Pan (a/b/g)(Phospho-Tyr55/53) | HDAC1 | HDAC1 (Phospho-Ser421) | Calmodulin | Calmodulin (Phospho-Thr79/Ser81) | Caspase 9 | Caspase 9 (Phospho-Ser196) | Catalase | Catalase (Phospho-Tyr385) | Chk1 | Chk1 (Phospho-Ser286) | FAK | FAK (Phospho-Ser910) | Smad1 | Smad1 (Phospho-Ser187) | Smad2 | Smad2 (Phospho-Ser467) | Smad2/3 (Phospho-Thr8) | Smad3 | Smad3 (Phospho-Ser213) | Caspase 1 | Caspase 1 (Phospho-Ser376) | MAP3K7/TAK1 | MAP3K7/TAK1 (Phospho-Thr184) | eNOS | eNOS (Phospho-Ser615) | PI3-kinase p85-alpha (Phospho-Tyr607) | PI3-kinase p85-subunit alpha/gamma |
| Abl1 | Abl1 (Phospho-Thr754/735) | B-RAF | B-RAF (Phospho-Ser446) | CaMK2-beta/gamma/delta | CaMK2-beta/gamma/delta (Phospho-Thr287) | PAK1 | PAK1 (Phospho-Ser204) | RSK1/2/3/4 | RSK1/2/3/4 (Phospho-Ser221/227/218/232) | Gab2 (Phospho-Tyr643) | LYN | LYN (Phospho-Tyr507) | PLCG1 | PLCG1 (Phospho-Tyr783) | CBL (Phospho-Tyr700) | FGFR1 | FGFR1 (Phospho-Tyr654) | Lamin A/C | Lamin A/C (Phospho-Ser392) | MITF | MITF (Phospho-Ser73) | PPAR-gamma | PPAR-gamma (Phospho-Ser112) | PP1 alpha | PP1 alpha (Phospho-Thr320) | Interferon-alpha/beta receptor alpha chain | Interferon-gamma receptor alpha (Phospho-Tyr457) | PDGFR beta | PDGFR beta (Phospho-Tyr1021) | FADD (Phospho-Ser194) | BLNK |
| BLNK (Phospho-Tyr96) | IL-10R-alpha | IL-10R-alpha (Phospho-Tyr496) | IL-4R/CD124 | IL-4R/CD124 (Phospho-Tyr497) | Bax | BAX (Phospho-Thr167) | PLK1 | PLK1 (Phospho-Thr210) | TGFBR1 | TGFBR2 | Fos | SREBP-1 | Caspase 3 | Beclin-1 | WNT1 | VDAC1 | COX41 | Beta actin | Negative control | Negative control | Negative control | Negative control | Empty | Empty | Empty | Empty | Empty | Empty | Empty | Empty | Position Marker |

**B**

| Control | +IL-1β +IL-23 | +IL-1β +IL-23+MA |
| --- | --- | --- |
| 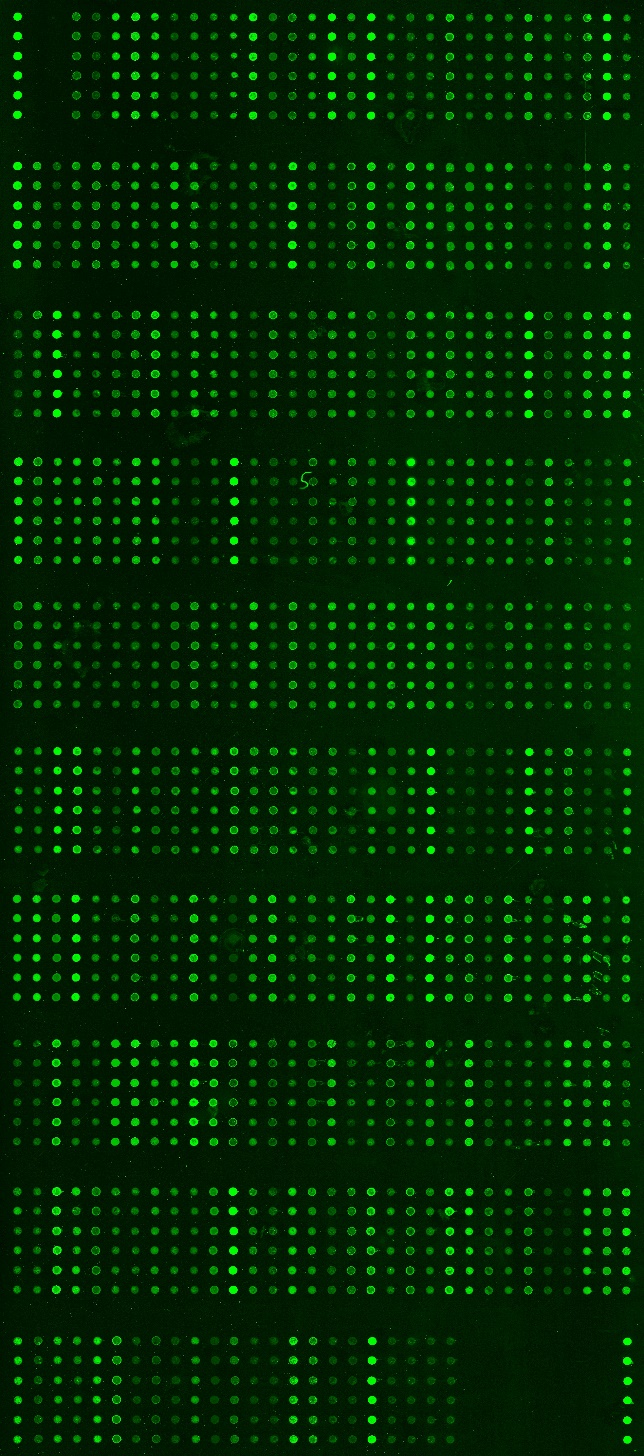 | 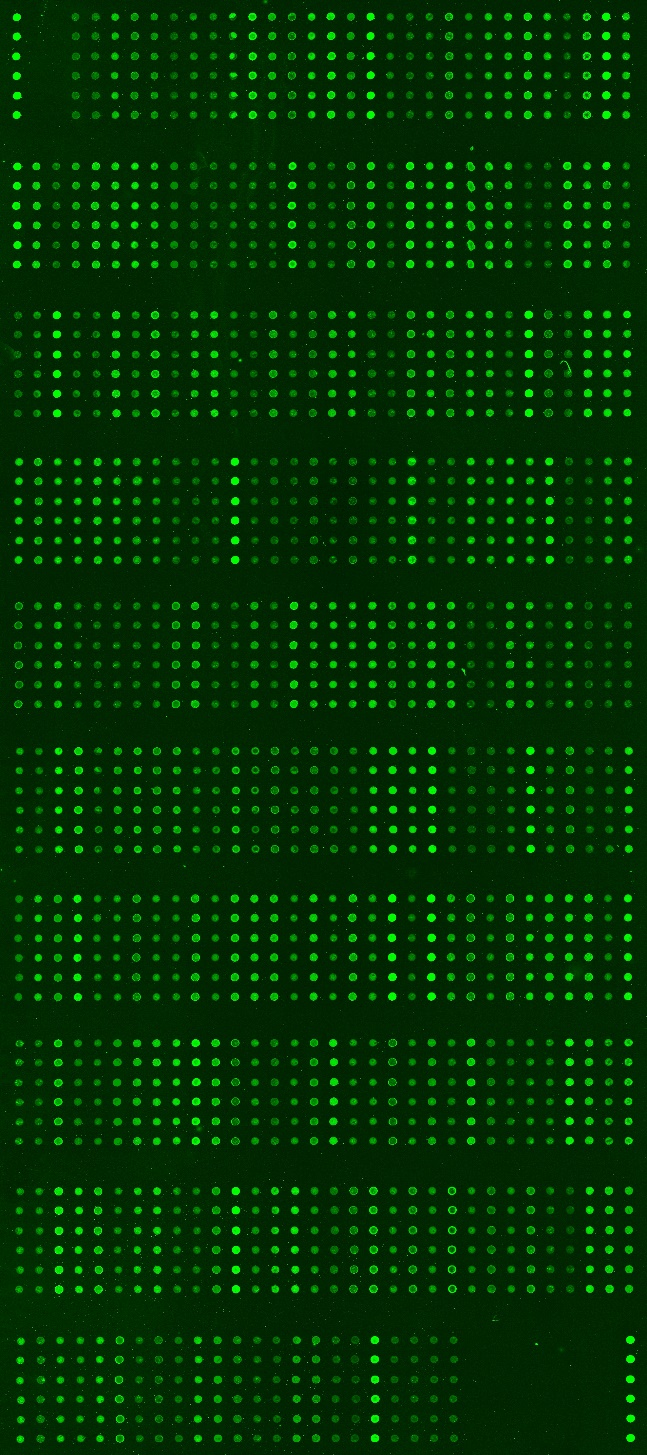 | 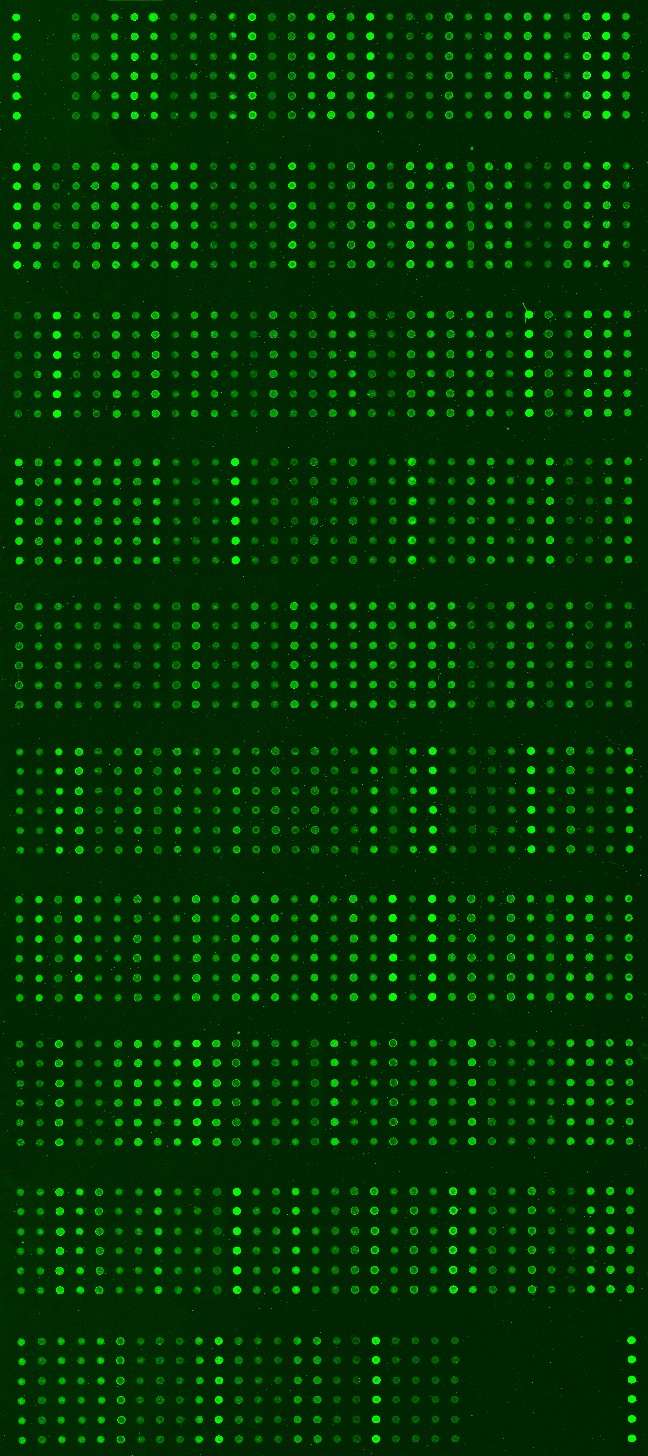 |
