# Supplementary material for: Inhibition of the activation of γδT17 cells through PPARγ–PTEN/Akt/GSK3β/NFAT pathway contributes to the anti-colitis effect of madecassic acid
Source: Cell Death Dis. 2020 Sep 14;11(9):752. doi: 10.1038/s41419-020-02969-x (PMC7490397; doi:10.1038/s41419-020-02969-x)
Supplement: Supplementary file 5 — Figure S5 [file 41419_2020_2969_MOESM5_ESM.docx]

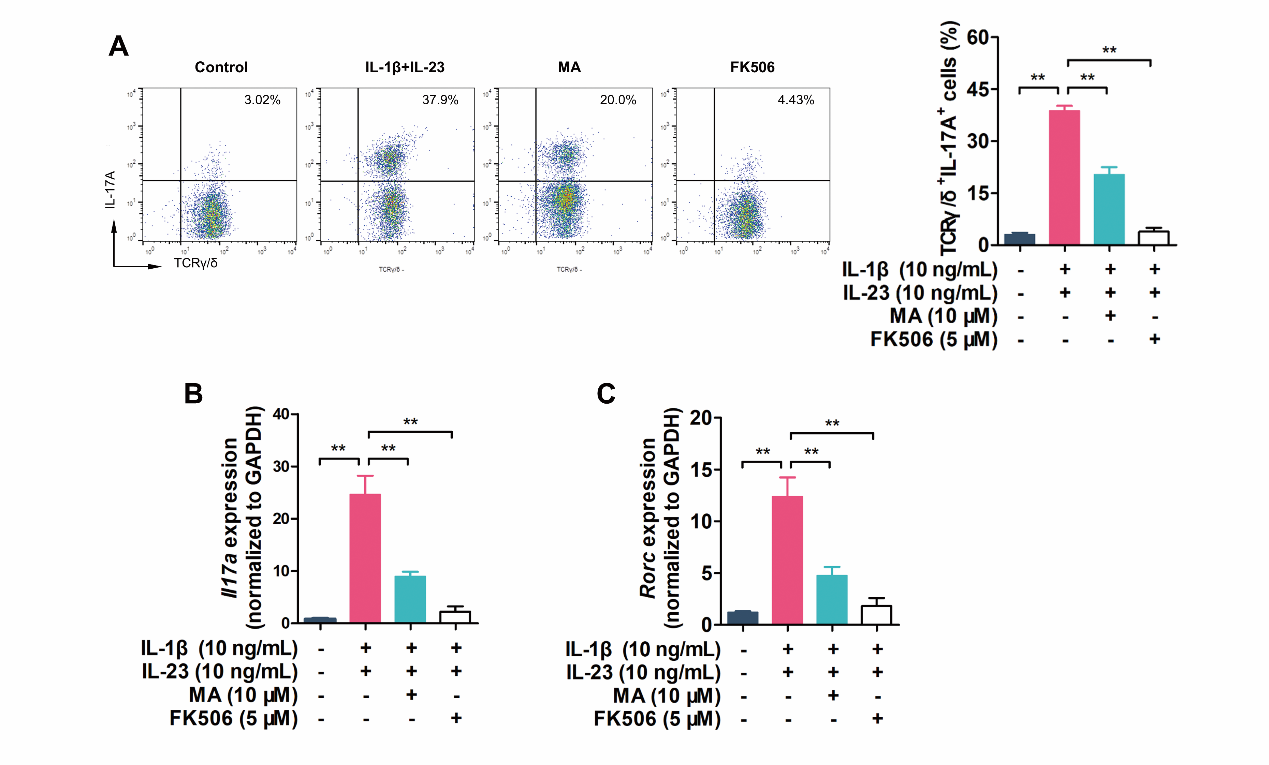


**Figure S5 FK506 inhibits the activation and the expression of *Il17a* and *Rorc in* the γδT17 cells.** The γδT cells were stimulated with IL-1β (10 ng/mL) and IL-23 (10 ng/mL) for 72 h in the presence or absence of madecassic acid (MA, 10 μM) or in combination with FK506 (5 μM). (A) The representative flow cytometry and percentages of IL-17A^+^ subpopulations present in the γδTCR^+^ T cells. (B&C) The expression levels of *Il17a* and *Rorc* as assessed by real-time PCR. The data are expressed as means ± SEM from 3 independent experiments. ***P*<0.01 *versus* indicated group.
